# Middle ear odontoma: A case report and review of the literature

**DOI:** 10.1016/j.ijscr.2020.01.050

**Published:** 2020-02-06

**Authors:** Ghaidaa Aljbli, Nouf Albakheet, Asem Alshawi, Yazeed Alshawi, Yaser Aljadhai, Ibrahim Shami

**Affiliations:** a Medical Intern at King Saud bin Abdulaziz University for Health Sciences, P.O. Box: 13214 Riyadh, Saudi Arabia; b Medical Intern at King Saud bin Abdulaziz University for Health Sciences, P.O.Box: 13247, Riyadh, Saudi Arabia; c Bachelor of Dental Surgery at NationalGuard Health Affairs, Riyadh, Saudi Arabia; d Otology Neurotology Fellow at King Abdullah Ear Specialist Center, Senior Registrar Otorhinolaryngologist at Prince Sultan Military Medical City, P.O.Box: 12233, Riyadh, Saudi Arabia; e Department of Neuroimaging and Intervention, Medical Imaging Administration, King Fahad, Medical City, Riyadh, Saudi Arabia; f Otorhinolaryngology and Head & Neck Surgery Section, Surgical Specialties Department, Main Hospital, King Fahad Medical City, P.O. Box. 59046, Riyadh 11525, Saudi Arabia

**Keywords:** Odontoma, Middle ear, Hearing loss, Conductive hearing loss, Facial nerve palsy

## Abstract

**Introduction:**

Odontomas are benign neoplasms originating from odontogenic tissues. Most of these masses are asymptomatic and can only be diagnosed correctly via radiological studies. Treatment of choice for management of odontoma involves surgical excision to prevent secondary complications and result in a favourable prognosis.

**Case presentation:**

This paper presents a rare case of odontoma of the middle ear in a four-year-old girl. The mass caused unilateral conductive hearing loss and facial nerve entrapment. We shed light on the typically vague clinical presentations of odontomas, as well as the available options in terms of diagnostic imaging modalities and their outcomes. Standard management of odontoma was followed to improve prognosis and avoid relapse. The aim of highlighting this case is to report on unique clinical, radiographical, and histological findings.

**Discussion:**

Retrotympanic odontomas are extremely rare. Only five cases in the English-language literature have investigated such an incident. All of the reviewed cases were asymptomatic and discovered only using radiological evidence. Two cases were managed surgically while the remainder were conservatively managed.

**Conclusion:**

In five of the six cases mentioned in the English-language literature (including this paper), odontomas occurred in the first decade of life, with an obvious female predilection. In one case, odontoma occurred in the third decade of life. Although odontoma is marked by its frequently benign origin, the absence of symptoms, and the necessity of surgical intervention, further studies are needed to analyse the different clinical pictures, and to allow these to add to what is currently known.

## Highlights

• Conductive hearing loss and facial nerve entrapment as consequences of Odontoma.
• CT scan and MRI are conclusive for the evaluation of odontoma lesions resembling numeral 3.
• Surgical removal is the standard of care in odontoma treatment.

## 1 Introduction

This work has been reported in line with the SCARE criteria [1]. Odontomas are slow-growing benign lesions of odontogenic origin composed of mesenchymal and epithelial dental tissues. Histologically, odontomas are formed by different dental elements, including enamel, cement, dentine, and, sometimes, pulp tissue [2,3]. Biologically, they are classified as “tooth hamartomas” with diverse degrees of differentiation: benign, localised, or aggressive malignant neoplasms that have high potential to metastasise [4]. Previous studies have discussed the pathogenesis of odontomas and their likely aetiologies, which include a history of trauma in the primary dentition, odontoblastic hyperactivity, genetic alterations during development, inflammatory and infectious processes, and hereditary anomalies such as Gardner’s syndrome and Hermann’s syndrome [5].

The development of retrotympanic odontomas is best accounted for by dental embryogenesis, particularly the dental lamina. The process of oral ectoderm thickening starts from the sixth week of gestation at the alveolar ridge of both the future maxilla and mandible. Simultaneously, the dental lamina proliferates and migrates posteriorly to outline the deciduous and permanent dentition, a process that ends by the age of four years. The eustachian tube, derived from the first pharyngeal pouch, may carry fragments of the posterior dental lamina to the developing middle ear cavity, giving rise to odontomas [6].

According to the World Health Organization (WHO), odontomas can be divided histologically into compound odontomas and complex odontomas. Compound odontomas consist of tooth-like structures arranged in typical patterns. Complex odontomas are made up of unorganized and mixed lesions of odontogenic epithelium, dentin, enamel, and its matrix, lacking the usual tooth-like structures associated with compound odontomas [7].

In the temporal bone, odontomas are usually asymptomatic; however, some patients experience ear pain and hearing deficits [8]. Overall, odontomas are primarily diagnosed in the first two decades of life based on incidental findings during routine radiological assessment, with female predilection. Radiologically, odontomas manifest as dense, radiopaque, and solid lesions filled with nodular elements and surrounded by fine, radiotransparent halos [[9], [10], [11]]. The recommended treatment is total surgical excision followed by histopathological studies to confirm the diagnosis [12].

The purpose of this study is to report a unique case of temporal odontoma with facial nerve compression. Similar cases found in the English-language literature are also reviewed.

## 2 Case presentation

A four-year-old female, medically free, presented at our centers otolaryngology clinic with a non-resolving facial palsy and ear discharge. The patient had a history of left facial nerve paralysis for more than six months accompanied by left ear yellowish foul-smelling discharge. No other aural complaints were identified, and the right ear was normal. Upon otoscopic examination, the left ear canal had an aural polyp with pus precluding full visualisation of the tympanic membrane. The right ear showed intact tympanic membrane and external auditory canal. The patient had left-sided lower motor neuron (LMN) facial nerve weakness, grade five on the House-Brackmann scale.

On further evaluation, conditioned play audiometry CPA using supra-aural headphones revealed hearing loss in the left ear mild to moderately severe and normal hearing sensitivity in the right ear. CPA with bone conduction testing resulted in masked bone conduction thresholds on the left ear within the normal range of 500–4,000 Hz, except for slight evidence of decreased hearing sensitivity at 2000 Hz. Auditory brainstem response confirmed the left-sided conductive hearing loss. Tympanometry revealed a type C tympanogram in the right ear, indicating negative air pressure in the middle ear. The left could not be tested due to active discharge. Bilaterally, the speech reception threshold SRT was consistent with pure tone averages.

**Fig. 1:**
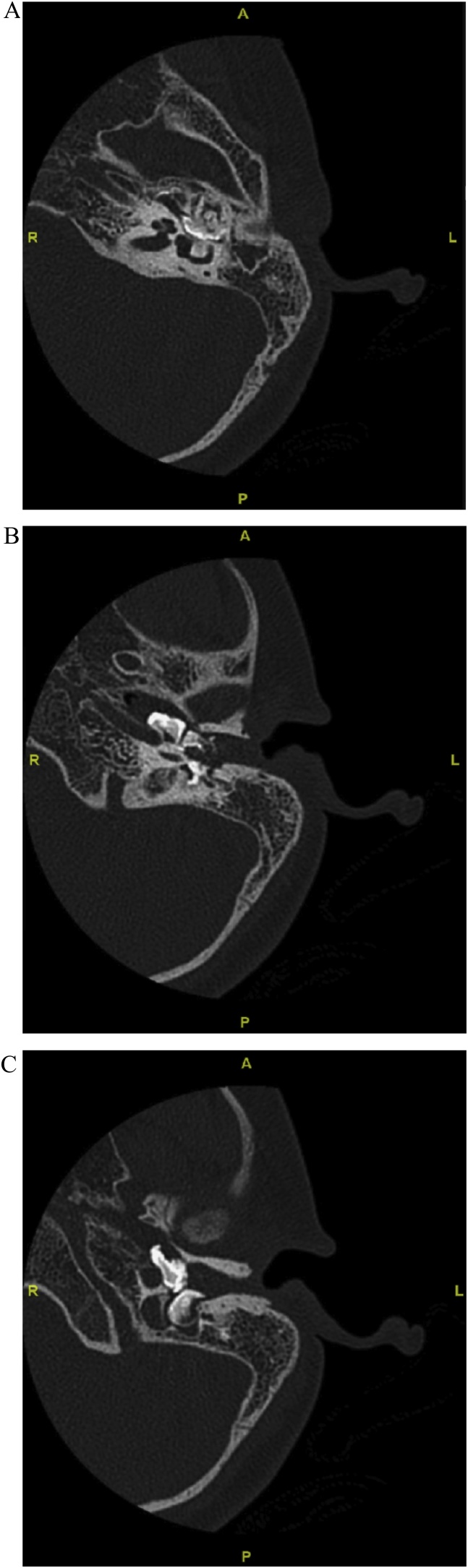
A. Computed tomography scan axial cut of the left mastoid area showing middle ear hyperdense mass compressing on tympanic segment of facial nerve and lateral semicircular canal. B. Lower down cut showing 2 odontomas, the anterior one in direct contact with carotid canal and eustachian tube. C. the lowest cut showing the same 2 odontomas one in contact with carotid artery while the posterior one compressing on the jaguar bulb.

A computed tomography (CT) scan revealed a lack of ossicles and three hyperdense calcified densities, almost filling the left middle ear cavity. This was consistent with the characteristics of odontomas. One of the densities was compressing the tympanic segment of the facial nerve and the lateral semi-circular canal. Another mass was anteriorly in direct contact with both the carotid canal and the eustachian tube, and posteriorly compressing on the jaguar bulb (Fig. 1A–C). Magnetic resonance imaging (MRI) revealed signs of left chronic suppurative otomastoiditis with no evidence of cholesteatoma.

After thorough counselling with the family, the patient was transferred to the operating theater for the following surgical interventions: left ear exploration with odontoma removal, decompression of the facial nerve’s tympanic segment, and cartilage tympanoplasty. Pre-operatively, prophylactic Cefazolin was administered to prevent operative infections. The patient was arranged in a supine position under general anaesthesia with endotracheal intubation. The procedure was undertaken in sterile conditions with continuous monitoring of the facial nerve.

**Fig. 2:**
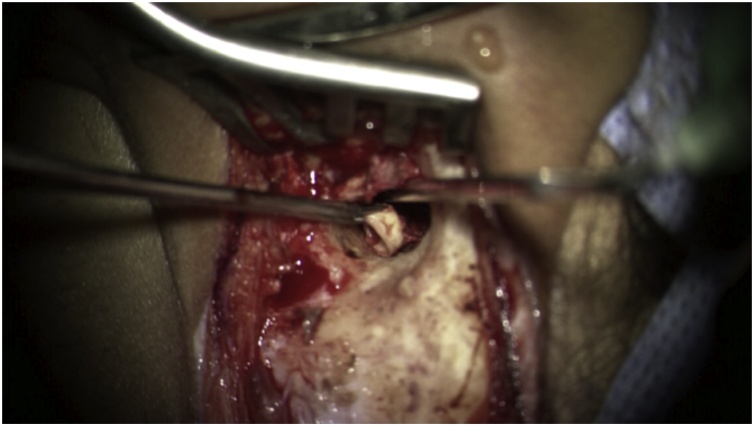
Intraoperative picture showing one of the odontomas while being removed from the middle ear.

Intra-operatively, through a retro-auricular incision, the operation started with periosteal flap elevation followed by anterior tympanotomy. This enabled the surgeon to lift the tympanomeatal flap. After initial ear exploration, examination of the middle ear cavity revealed keratinised tissues and a firm fibrotic aural polyp. These extruded into the external auditory canal, covering the odontoma, and were initially cauterised and debrided. No ossicles or stapes suprastructure were observed as they appeared to have been eroded by the odontoma. However, the head of stapes was noticed. After polyp removal, canaloplasty, and atticotomy, the surgical team noticed three embedded teeth surrounded by a fibrotic soft tissue. The first tooth, found in the attic, was compressing the tympanic segment of the facial nerve. This was fully drilled to decompress the facial nerve. The second tooth, covering the promontory and extending into the hypotympanum, was extracted completely (Fig. 2). Once we reached the jugular vein, the third tooth was found occupying the eustachian tube orifice and firmly attached to the carotid canal. This tooth was kept in place since opening the eustachian tube could have led to carotid artery injury. After removing most of the odontoma, the facial nerve’s swollen tympanic segment was fully exposed, and its sheath was incised to relieve the edema. Finally, the attic and the perforated tympanic membrane were reconstructed using temporalis facial and conchal cartilage.

The patient tolerated the surgery well. Post-operatively, on day one, the patient did not present with pain complaints. During examination, she was vitally stable, with no active bleeding or oozing on the left ear. In addition, the Weber test was lateralised to the operated ear. The patient was discharged on oral Cefuroxime for one week. At six-month follow-up, the patient’s condition was stable with no deterioration, relapse, or active complaints. Moreover, the patient noted improvement in her facial palsy, which was apparent on examination (grade three left facial palsy on the House-Brackmann scale). Ear evaluation by otoscopy showed bilateral intact tympanic membranes. Tuning fork examination on the operated ear demonstrated negative Rinne and lateralisation during the Weber test. Audiological tests reported the same mild to moderately severe conductive hearing loss on the left side. These results were expected, and so a hearing restoration procedure was planned for the second stage of the intervention.

## 3 Discussion

Despite the wealth of case reports concerning odontomas in tooth-bearing areas at the head and neck levels, a review of the literature revealed only five case reports of temporal bone odontomas. The first case was reported in 1975 and the last case was reported in 2014. To the best of our knowledge, no cases have been documented in the literature since 2014.

The first case of middle ear odontoma was described in 1975 by Bellucci et al., who reported a seven-year-old boy admitted to hospital due to right-sided conductive hearing loss, episodic pain, and recurrent upper respiratory tract infection (URTI). Dental examination reported normal dentation except for a left sided missing lower bicuspid tooth. Physical examination of the right ear revealed a dull whitish tympanic membrane with lack of apparent landmarks. Radiography confirmed the diagnosis of odontoma. Exploratory tympanotomy on the right ear showed a hard, immobile mass palpated retro-tympanically. Bellucci et al. decided to treat the patient conservatively without any surgical intervention to preserve cochlear and facial nerve functions [13].

In 2004, Sun et al. reported the results of a twenty-five-year follow-up with the patient treated by Bellucci et al., who had been experiencing long-standing hearing loss. Otoscopy was unremarkable, yet audiometry showed deterioration with severe progressive mixed hearing loss on the right side and mild sensorineural hearing loss in the left ear. A CT scan revealed hyperdensity on the surface of the right middle ear promontory, apparent as one-millimeter thick wavy stripes, resembling a backward numeral “3”. The decision was made to continue with conservative management and to offer the patient BiCROSS (bilateral contralateral routing of signal) [14].

In 1981, McClatchey et al. reported a four-year-old girl with a history of right ear infection, otorrhea, and hearing loss (40 dB). Cholesteatoma was diagnosed clinically and radiologically with a mass occupying the posterior mesotympanum and was chosen for surgical intervention. A posterior bony canal dehiscence with mass protruding laterally to the annulus tympanicus in the middle ear which was successfully managed and excised. Histopathology came as tissue fragment mixture containing cementum, dentin, dental follicles, and proliferating dental lamina, all of which are typical for compound odontoma [15].

In 1991, Prasad et al. reported two cases of retrotympanic odontoma. One case was a six-year-old girl who presented with left ear pain and a history of myringotomy with failed tube insertion. The patient displayed a left sided tympanic membrane retraction and conductive hearing loss (50 dB). Exploratory tympanotomy revealed a hard-bony lesion occupying the oval window area with accompanying congenital ossicular abnormalities and lack of stapes. Ossicles were firmly attached to malleus and hence removed. The procedure was aborted and followed by a left graft tympanoplasty resulting in no subsequent change in hearing. Twelve years later, the patient reappeared complaining of left-sided otorrhea. Examination and audiogram results were similar to those conducted previously. Imaging revealed multiple calcified masses at the mesotympanum and protympanum, one of which contained cementum, dentin, and pulp cavity. Surgical exploration showed a mass obscuring the tympanic facial nerve and oval window with extensive cholesteatoma of the middle ear cleft. The surgical team removed the tooth impacted in the protympanum; however, the patient's condition necessitated a radical mastoidectomy eventually.

The second case reported by Prasad et al. is similar to the first reported case in 1975. A thirty-five-year-old female patient presented with mixed hearing loss. CT scan demonstrated a calcified mass, with cementum and dentin, embedded within the otic capsule in the middle ear space. To preserve proper cochleovestibular functioning, they decided to conservatively manage her [16].

Lastly, in 2014, a study reported an infant with hypoplastic left auricle, right ear atresia, and right-sided LMN facial palsy with no dysmorphic features. ABR displayed irresponsive right ear and moderate conductive hearing loss in the left ear that was managed with hearing aids.

CT scan showed a complete external auditory canal atresia and opacified right middle ear cavity and mastoid space. Right mastoid space was filled with a well-defined transparent lesion consistent with complex odontoma. Right facial nerve was hypoplastic with a small bony facial canal. Decision for surgical intervention was deferred until the patient becomes older [17].

## 4 Conclusion

With only a handful of cases reported in the literature, odontomas of the middle ear cleft are extremely rare. These lesions are usually asymptomatic and recognised during routine diagnostic studies. Given that odontomas are considered benign lesions, surgical management is generally limited to symptomatic patients, depending on the degree of hearing impairment and facial nerve involvement. However, if feasible, odontomas should be excised to prevent secondary complications, to obtain histopathological diagnosis, and to ensure optimal patient outcomes. This case report emphasises the diverse clinical, radiographical, and histological findings that are possible for retrotympanic odontomas. It also illustrates the potential for improvement in long-standing facial nerve paralysis with nerve decompression in patients suffering from facial nerve compressing odontomas.

## Funding

No source of funding.

## Ethical approval

IRB approval has been guaranteed by King Fahad Medical City. IRB Log number: 18-684.

## Consent

Parental consent on behalf of the patient had been obtained and available upon request.

## Author contribution

Ghaidaa Aljbli has contributed in writing the introduction, case, and discussion.

Nouf Albakheet has contributed in writing the introduction, case, and discussion.

Asem Alshawi has contributed in writing the case.

Yazeed Al-shawi has contributed in writing the case.

Yaser Aljadhai has contributed in writing the case.

Ibrahim Shami has contributed as the supervisor.

## Registration of research studies

No human participation.

## Guarantor

Dr. Yazeed Al-shawi.

## Provenance and peer review

Not commissioned, externally peer-reviewed.

## Declaration of Competing Interest

No conflict of interest.
